# Morbidity and Mortality of Very-Low-Birth-Weight Preterm Neonates in a Tertiary Neonatal Intensive Care Unit in Northeastern Mexico: A Five-Year Retrospective Cohort Study

**DOI:** 10.3390/pediatric18040095

**Published:** 2026-07-11

**Authors:** Esteban López-Garrido, Alejandra Guadalupe Polina Lugo, Ana Patricia Ortega-González, Hadassa Yuef Martínez-Padrón

**Affiliations:** 1 Unidad de Cuidados Intensivos en Neonatos, Hospital Regional de Alta Especialidad Ciudad Victoria, Servicios de Salud del Instituto Mexicano del Seguro Social Para el Bienestar (IMSS-BIENESTAR), Libramiento Guadalupe Victoria S/N, Área de Pajaritos, Victoria 87087, Tamaulipas, Mexico; estebanlopez_garrido@hotmail.com; 2 Facultad de Medicina, Universidad Autónoma de Tamaulipas, Matamoros 87300, Tamaulipas, Mexico; alejandra_pl95@hotmail.com (A.G.P.L.); patriciaortega2607@gmail.com (A.P.O.-G.); 3 Subdirección de Enseñanza e Investigación, Hospital Regional de Alta Especialidad Ciudad Victoria, Servicios de Salud del Instituto Mexicano del Seguro Social Para el Bienestar (IMSS-BIENESTAR), Libramiento Guadalupe Victoria S/N, Área de Pajaritos, Victoria 87087, Tamaulipas, Mexico

**Keywords:** prematurity, very low birth weight, neonatal morbidity, neonatal mortality, neonatal intensive care unit, preterm infant

## Abstract

Background: Prematurity remains a major global health challenge and is a leading cause of neonatal morbidity and mortality worldwide. The risk of adverse outcomes is inversely associated with gestational age and birth weight. Although advances in neonatal intensive care have improved survival rates over recent decades, very-low-birth-weight (VLBW) preterm neonates continue to experience substantial morbidity and remain vulnerable to long-term complications. Objective: This study aimed to evaluate the morbidity and mortality of very-low-birth-weight preterm neonates (<1500 g) admitted to the Neonatal Intensive Care Unit (NICU) of the Hospital Regional de Alta Especialidad de Ciudad Victoria (HRAEV), Mexico. Materials and Methods: A retrospective observational cohort study was conducted through a review of medical records of VLBW preterm neonates admitted to the NICU between January 2019 and December 2023. Demographic, perinatal, clinical, and outcome-related data were collected and analyzed. Results: A total of 58 VLBW preterm neonates were included. Mean gestational age was 29.8 ± 2.7 weeks, and mean birth weight was 1109 ± 238 g. The most common morbidities were respiratory distress syndrome (81.0%), apnea of prematurity (72.4%), hyperbilirubinemia (68.9%), pneumonia (34.5%), sepsis (32.7%), patent ductus arteriosus (25.8%), bronchopulmonary dysplasia (26.9% among infants who survived to 36 weeks’ PMA), necrotizing enterocolitis (15.5%), and intraventricular hemorrhage (12.0%) and retinopathy of prematurity (8.6%). Overall mortality was 10.3%. Conclusions: VLBW preterm neonates remain at high risk for significant morbidity despite relatively favorable survival rates. Respiratory distress syndrome, apnea of prematurity, hyperbilirubinemia, and sepsis were the most frequent complications, whereas deaths occurred mainly in the context of severe respiratory and systemic complications, including neonatal asphyxia, pulmonary hypertension, sepsis, shock, and multiple organ failure. Survival outcomes should be interpreted cautiously because of differences in study populations, referral patterns, local viability practices, and study design across neonatal settings.

## 1. Introduction

Prematurity and very low birth weight (VLBW, <1500 g) remain major global health challenges and are among the leading causes of neonatal morbidity and mortality worldwide [1,2]. Despite substantial advances in neonatal intensive care, the global incidence of preterm birth has shown only modest reductions over the last decade, with an estimated 13.4 million preterm births reported in 2020 [1,2]. Prematurity-related complications accounted for approximately 900,000 deaths among children under five years of age in 2019 [1,2].

The risk of adverse neonatal outcomes is strongly associated with gestational age and birth weight [3,4]. Although VLBW infants represent a relatively small proportion of all live births, they account for a disproportionate number of neonatal deaths, prolonged hospitalizations, and long-term neurodevelopmental impairments [3,5]. Mortality rates among VLBW neonates vary considerably across regions, ranging from less than 10% in high-income countries to more than 30% in resource-limited settings [5,6]. Common morbidities in this population include respiratory distress syndrome (RDS), neonatal sepsis, patent ductus arteriosus (PDA), bronchopulmonary dysplasia (BPD), necrotizing enterocolitis (NEC), intraventricular hemorrhage (IVH), and retinopathy of prematurity (ROP) [4,5,6].

International studies have demonstrated substantial improvements in survival among VLBW infants over recent decades [5,7,8]. However, important disparities persist between countries and neonatal networks [5,9,10,11]. Factors such as gestational age, birth weight, availability of neonatal intensive care resources, antenatal care, infection-control practices, and perinatal management significantly influence neonatal outcomes [5]. Despite advances in neonatal care, sepsis, respiratory complications, necrotizing enterocolitis, pulmonary hemorrhage, and severe neurological injury continue to be among the leading causes of mortality and long-term disability in this population [9,11,12,13,14].

Large neonatal networks, including those from Japan, Europe, North America, and South America, have reported progressive reductions in mortality and severe morbidity among VLBW infants [4,5,12,13,14]. Nevertheless, survival and morbidity outcomes remain highly heterogeneous across healthcare systems and geographic regions [5,12]. These findings underscore the need for ongoing regional surveillance and outcome assessment to identify opportunities for quality improvement in neonatal care [12,13,14].

In Mexico, VLBW infants account for approximately 1–2% of all live births [3]. Previous Mexican studies conducted in tertiary referral centers have reported survival rates ranging from approximately 84% to 92%. However, survival estimates vary considerably across studies because of differences in patient characteristics, birth weight and gestational age criteria, healthcare resources, and study periods [10,15,16]. However, information regarding morbidity patterns, mortality causes, and clinical outcomes among VLBW infants remains limited in several regions of the country, particularly in northeastern Mexico [10,15,16].

Therefore, the present study aimed to evaluate the short-term morbidity and mortality profile of very-low-birth-weight (VLBW) preterm neonates admitted to the Neonatal Intensive Care Unit (NICU) of a tertiary referral hospital in northeastern Mexico during a five-year period. Specifically, we sought to describe major in-hospital neonatal morbidities, respiratory support requirements, surgical interventions, length of hospital stay, and mortality before hospital discharge. Given the limited availability of regional data, these findings may contribute to a better understanding of short-term clinical outcomes among VLBW infants in northeastern Mexico and help identify opportunities for quality improvement in neonatal care.

## 2. Materials and Methods

### 2.1. Study Design and Setting

This retrospective observational cohort study was conducted at the Neonatal Intensive Care Unit (NICU) of the Hospital Regional de Alta Especialidad de Ciudad Victoria (HRAEV), a tertiary referral center in northeastern Mexico. HRAEV is a tertiary referral hospital that receives high-risk pregnancies and neonatal transfers from multiple hospitals across the state of Tamaulipas and neighboring regions. Most high-risk pregnancies are transferred antenatally, allowing delivery to occur at our institution; consequently, the majority of VLBW infants are inborn, whereas fewer than 30% are referred after birth from other institutions. The NICU provides care for both inborn and outborn neonates requiring specialized neonatal intensive care. Medical records of very-low-birth-weight (VLBW) preterm neonates admitted between 1 January 2019 and 31 December 2023 were reviewed.

Nutritional management in the NICU generally included early parenteral nutrition, initiation of trophic enteral feeding when clinically feasible, and progressive advancement of enteral feeds according to institutional protocols and patient tolerance. Mother’s own milk was the preferred source of enteral nutrition whenever available.

Patient identification was performed through NICU admission and discharge registries. Subsequently, electronic and paper-based medical records were reviewed to obtain demographic, perinatal, clinical, therapeutic, and outcome-related information. Data were collected using a standardized case report form specifically designed for this study.

### 2.2. Study Population and Eligibility Criteria

The study population consisted of preterm neonates with a birth weight < 1500 g admitted to the NICU during the study period.

Inclusion criteria:

- Gestational age < 37 weeks.
- Birth weight < 1500 g.
- Admission to the NICU during the study period.

Exclusion criteria:

- Neonates who did not meet the predefined gestational age or birth weight criteria.
- Referred neonates with insufficient clinical information for outcome assessment.
- Only one referred neonate who died during the first hour after admission was excluded because complete clinical information was unavailable for analysis.
- Infants who died before 36 weeks PMA were excluded from BPD assessment.

Elimination criteria:

- Missing medical records.
- Incomplete clinical records preventing collection of study variables.

### 2.3. Sample Size and Sampling Strategy

**Figure 1:**
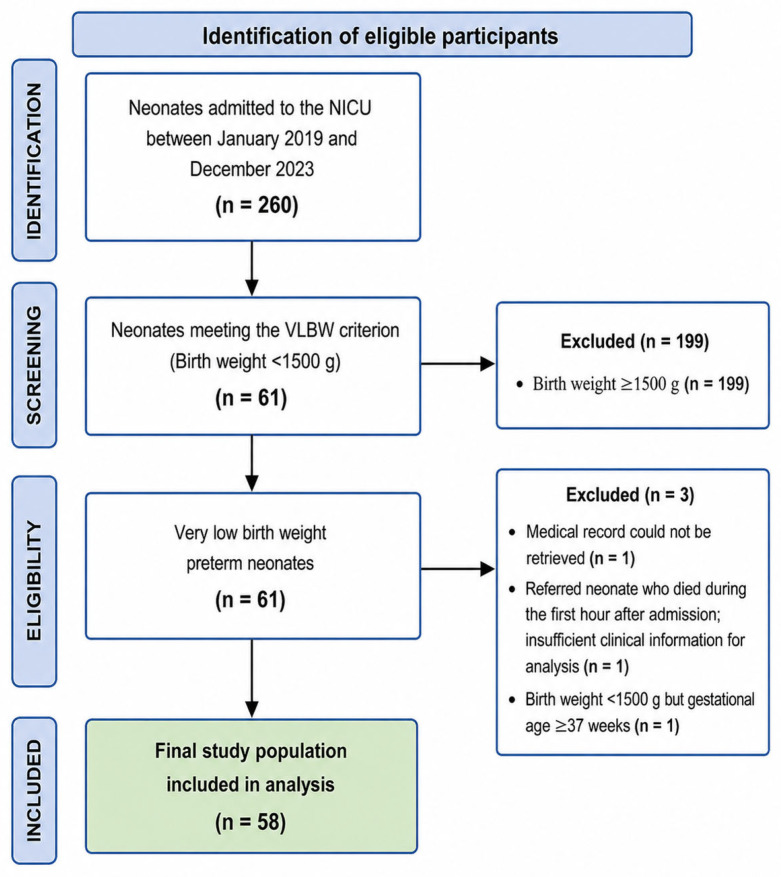
Flow diagram of patient selection, inclusion, and final study population.

Because of the retrospective design, a non-probabilistic consecutive sampling strategy was employed. All eligible VLBW preterm neonates admitted during the study period were included. Fifty-eight neonates fulfilled the selection criteria and constituted the final study population (Figure 1).

### 2.4. Data Collection and Clinical Variables

The following variables were collected: gestational age, birth weight, sex, Apgar scores, maternal age, antenatal corticosteroid administration, type of pregnancy, mode of conception, mode of delivery, surfactant administration, duration of invasive and non-invasive respiratory support, cumulative oxygen therapy, length of hospital stay, neonatal morbidities, survival status at discharge, and cause of death when applicable.

### 2.5. Definitions of Clinical Outcomes

Respiratory distress syndrome (RDS) was diagnosed according to compatible clinical manifestations, including tachypnea, grunting, chest retractions, and increased oxygen requirements, together with characteristic radiographic findings and the need for respiratory support and/or surfactant administration [17].

Apnea of prematurity was defined as a respiratory pause lasting ≥20 s or a shorter pause associated with bradycardia and/or oxygen desaturation in the absence of another identifiable cause [18].

Neonatal sepsis was defined as a culture-confirmed or clinically suspected infection requiring antimicrobial treatment. Early-onset sepsis was defined as occurring within the first 72 h of life, whereas late-onset sepsis (LOS) was defined as occurring after 72 h of life [19].

Patent ductus arteriosus (PDA) was diagnosed by echocardiography performed by a pediatric cardiologist and classified according to hemodynamic significance [20].

Bronchopulmonary dysplasia (BPD) was defined as the requirement for supplemental oxygen and/or respiratory support at 36 weeks of postmenstrual age, according to the National Institutes of Health consensus definition [21].

Necrotizing enterocolitis (NEC) was diagnosed and staged according to the modified Bell criteria [22].

Intraventricular hemorrhage (IVH) was classified according to the Papile grading system based on cranial ultrasonography findings [23].

Retinopathy of prematurity (ROP) was diagnosed and classified according to the International Classification of Retinopathy of Prematurity (ICROP), Third Edition [24].

Pulmonary hypertension was diagnosed by echocardiographic evidence of elevated pulmonary arterial pressure and/or right ventricular pressure overload [25].

Mortality was defined as death occurring during hospitalization before discharge from the NICU. Length of hospital stay was calculated as the number of days from birth until discharge or death.

Hyperbilirubinemia was defined as jaundice requiring phototherapy and/or exchange transfusion according to age-specific serum bilirubin thresholds.

Pneumonia was diagnosed according to compatible clinical manifestations and radiographic findings suggestive of pulmonary infection, with or without microbiological confirmation.

### 2.6. Statistical Analysis

Descriptive statistics were used to summarize demographic and clinical characteristics. Normality of continuous variables was assessed using the Shapiro–Wilk test. Variables with a normal distribution are presented as mean ± standard deviation (SD), whereas non-normally distributed variables are reported as median and interquartile range (IQR). Categorical variables are expressed as frequencies and percentages. Because only six deaths occurred during the study period, multivariable modeling was not performed to avoid overfitting. Similarly, effect size estimates, including odds ratios and confidence intervals, were not calculated because of the limited number of mortality events and the resulting instability of estimates.

For subgroup analyses, percentages were calculated using the number of patients within each gestational age, birth weight, or annual cohort category as the corresponding denominator. Comparisons between survivors and non-survivors were performed using Student’s *t*-test or the Median test for continuous variables, as appropriate. Categorical variables were compared using Fisher’s exact test or the Chi-square test. Given the limited number of deaths observed in the cohort, these analyses should be interpreted as exploratory. Statistical significance was established at a two-sided *p*-value < 0.05.

All analyses were performed using IBM SPSS Statistics version 26.0 (IBM Corp., Armonk, NY, USA).

### 2.7. Ethical Considerations

The study was conducted in accordance with the Declaration of Helsinki and national regulations governing research involving human subjects. The investigation consisted exclusively of a retrospective review of clinical records obtained during routine medical care, and no additional procedures, interventions, or biological samples were collected for research purposes.

Patient confidentiality was preserved through anonymization of all data before analysis. Access to medical records was authorized by the institutional research committee. Due to the retrospective nature of the study and the use of anonymized data, the requirement for informed consent was waived according to institutional regulations.

## 3. Results

During the study period (January 2019–December 2023), 260 neonates were admitted to the Neonatal Intensive Care Unit (NICU). Among them, 61 (23.5%) met the birth weight criterion for very low birth weight (VLBW, <1500 g). After application of the eligibility criteria, 58 VLBW preterm neonates were included in the final analysis (Figure 1).

The baseline demographic and clinical characteristics of the study population are summarized in Table 1 and Table 2. The mean gestational age was 29.8 ± 2.7 weeks, and the mean birth weight was 1109 ± 238 g. Females accounted for 55.2% of the cohort, whereas males represented 44.8%. Median Apgar scores were 7 (IQR, 5–8) at 1 min and 8 (IQR, 7–9) at 5 min. Cesarean delivery was performed in 96.6% of pregnancies, and antenatal corticosteroids were administered to 56.9% of mothers. The median duration of invasive mechanical ventilation was 1 day (IQR, 0–6), while the median duration of non-invasive ventilation was 2 days (IQR, 0–4). The median cumulative duration of oxygen therapy was 9.5 days (IQR, 1.7–30), and the median length of hospital stay was 51.5 days (IQR, 36–71.75) (Table 1).

Maternal age averaged 30.1 ± 8.8 years. Most mothers had completed a university education (48.3%), and 41.4% were in a common-law union. Hypertensive disorders of pregnancy, including preeclampsia, represented the most common maternal comorbidity (31.0%), followed by gestational diabetes (7.0%), SARS-CoV-2 infection (5.0%), and systemic lupus erythematosus (1.7%). Multiple gestations accounted for 36.2% of pregnancies, whereas 20.7% resulted from assisted reproductive technologies (Table 2).

Respiratory morbidity represented the predominant clinical burden in this cohort. Respiratory distress syndrome (RDS) was the most common complication, affecting 81.0% of neonates, followed by apnea of prematurity (72.4%), hyperbilirubinemia (68.9%), pneumonia (34.5%), sepsis (32.7%), patent ductus arteriosus (25.8%), bronchopulmonary dysplasia observed in 14 infants, representing 26.9% of those who survived to 36 weeks’ PMA, necrotizing enterocolitis (15.5%), intraventricular hemorrhage (12.0%), and retinopathy of prematurity (8.6%) (Table 3 and Table 4). Respiratory and infectious complications were concentrated among the most immature infants, particularly those born before 29 weeks of gestation. The highest frequencies of RDS, sepsis, and mortality were observed among neonates born before 29 weeks of gestation and among those weighing less than 1000 g at birth.

Regarding fetal growth patterns, 14 neonates (24.1%) were classified as small for gestational age, whereas seven infants (12.1%) had a documented diagnosis of intrauterine growth restriction (IUGR). Detailed anthropometric data required to distinguish between symmetric and asymmetric growth restriction were not consistently available in the medical records.

Mechanical ventilation was required in 62.0% of neonates, whereas 70.7% received non-invasive respiratory support. Among neonates diagnosed with patent ductus arteriosus, pharmacological treatment was administered in 11 cases, resulting in successful ductal closure in 73% of treated patients. Two neonates required surgical ligation, and one underwent interventional closure.

Necrotizing enterocolitis (NEC) was diagnosed in nine neonates according to modified Bell criteria. Three infants developed complicated NEC (Bell stage III) requiring surgical intervention, including laparotomy and ileostomy. Six additional infants were classified as suspected NEC (Bell stage IA). Two of these infants underwent surgery because of persistent subocclusive intestinal symptoms attributed to intestinal immaturity rather than confirmed NEC, whereas the remaining four cases were managed medically. Overall, six abdominal surgical procedures were performed during hospitalization, including five laparotomies and one ileostomy. Three procedures were performed for complicated NEC, two for subocclusive episodes associated with intestinal immaturity that interfered with enteral feeding and were classified as suspected intestinal atresia, and one corresponded to primary closure of gastroschisis.

The overall mortality was 10.3% (6/58). Mortality occurred across different gestational age and birth weight categories. Because only six deaths were observed, the study was not powered to identify reliable mortality patterns according to gestational age or birth weight, and these findings should therefore be interpreted descriptively. The principal causes of death were neonatal asphyxia, cardiogenic shock, neurogenic shock, pulmonary hypertension, severe abdominal sepsis, and multiple organ failure. Contributing underlying conditions included placental abruption, disseminated intravascular coagulation with pulmonary hemorrhage, severe respiratory distress syndrome, complicated NEC, and septic shock. The median length of hospital stay was significantly shorter among non-survivors than among survivors (2.0 days [IQR, 1.0–13.5] vs. 58.5 days [IQR, 42.7–74.7]; *p* = 0.010), reflecting the occurrence of death during the early neonatal period. Among the 52 survivors, median discharge weight was 2180 g (IQR 1980–2450), and median postmenstrual age at discharge was 37.8 weeks (IQR 36.4–39.5).

When annual morbidity frequencies were analyzed according to year of admission, most complications remained relatively stable throughout the study period (Table 5). Sepsis was observed in 7 of 11 infants admitted in 2022 (63.6%), representing the highest annual frequency and the only morbidity showing statistically significant variation across years (*p* = 0.014). However, given the small annual sample sizes and the multiple comparisons performed, this finding should be interpreted cautiously and considered exploratory and hypothesis-generating rather than definitive. No significant annual variations were observed for respiratory distress syndrome, apnea of prematurity, hyperbilirubinemia, patent ductus arteriosus, bronchopulmonary dysplasia, necrotizing enterocolitis, intraventricular hemorrhage, or retinopathy of prematurity. The annual distribution of VLBW preterm neonates included in the study was as follows: 16 infants in 2019, 8 in 2020, 11 in 2021, 11 in 2022, and 12 in 2023.

Comparison of clinical and demographic characteristics between survivors and non-survivors is presented in Table 6. Although survivors tended to have a higher gestational age and birth weight than non-survivors, these differences did not reach statistical significance. Similarly, no significant associations with mortality were observed for sex, antenatal corticosteroid exposure, surfactant administration, type of pregnancy, or mode of delivery. Non-survivors and survivors had similar median Apgar scores at 5 min (8 [IQR 4.5–9] vs. 8 [IQR 8–9]; *p* = 0.493). Conversely, the median length of hospital stay was significantly shorter among non-survivors than survivors (2 [IQR 1–13.5] vs. 58.5 [IQR 42.7–74.7] days; *p* = 0.010). When annual morbidity frequencies were analyzed, only sepsis showed statistically significant differences across years of admission (*p* = 0.014), whereas no significant annual variations were observed for the remaining morbidities (*p* > 0.05).

## 4. Discussion

This study describes the morbidity and mortality profile of very-low-birth-weight (VLBW) preterm neonates admitted to a tertiary neonatal intensive care unit in northeastern Mexico over a five-year period. The overall mortality rate was 10.3%. Although this estimate appears lower than that reported in several Latin American cohorts and falls within the range described in some contemporary neonatal networks, direct comparisons should be interpreted cautiously because of differences in patient characteristics, healthcare resources, local viability practices, referral patterns, and study design [4,5,9,10,13,14,18,19,20]. Moreover, the relatively small sample size and limited number of deaths reduce the precision of mortality estimates. Nevertheless, the present findings provide valuable regional information regarding the burden of prematurity-associated morbidity in a tertiary referral setting.

Respiratory morbidity represented the principal clinical burden in this population. Respiratory distress syndrome (RDS) affected more than four-fifths of neonates and was the most common complication, followed by apnea of prematurity and hyperbilirubinemia. Similar morbidity patterns have been reported in cohorts from Japan, Taiwan, Australia, and South America, where respiratory complications remain a major determinant of NICU admission, prolonged hospitalization, and healthcare utilization among VLBW infants [4,5,6,7,8,18,19,20]. The high frequency of RDS observed in our study is likely related to pulmonary immaturity secondary to low gestational age and birth weight, both of which remain the strongest predictors of respiratory morbidity in preterm neonates [4,5,6]. Furthermore, respiratory morbidity tended to decrease with increasing gestational age, supporting the well-established association between pulmonary immaturity and adverse respiratory outcomes among VLBW infants.

Antenatal corticosteroid administration is one of the most effective interventions for improving neonatal outcomes, reducing respiratory distress syndrome, intraventricular hemorrhage, and mortality. Although 56.9% of mothers in our cohort received antenatal corticosteroids, this proportion remains below that reported by several contemporary neonatal networks, where coverage frequently exceeds 80% [4,5,18,19,20]. Increasing antenatal corticosteroid utilization may therefore represent a feasible strategy to further improve neonatal outcomes. Likewise, the need for surfactant therapy in more than half of the cohort underscores the substantial respiratory support requirements of this highly vulnerable population.

Mechanical ventilation remains an essential component of neonatal intensive care; however, prolonged ventilatory support has been consistently associated with chronic lung injury and bronchopulmonary dysplasia. In our cohort, invasive mechanical ventilation was required in nearly two-thirds of neonates, with a median duration of 1 day (IQR, 0–6). Although the duration of invasive ventilation was relatively short for most infants, bronchopulmonary dysplasia occurred in 26.9% of those who survived to 36 weeks’ postmenstrual age, predominantly among the most immature neonates, consistent with previous reports. Although no statistically significant association with mortality was identified, bronchopulmonary dysplasia affected approximately one-quarter of patients and occurred predominantly among the most immature infants, consistent with previous studies demonstrating the strong association between extreme prematurity, prolonged respiratory support, and chronic pulmonary morbidity [15,21].

The incidence of patent ductus arteriosus and intraventricular hemorrhage was comparable to that reported in other VLBW populations [4,6,15,18,19,20]. Most PDA cases were successfully managed pharmacologically, whereas severe neurological complications remained relatively uncommon. Similarly, retinopathy of prematurity affected fewer than 10% of neonates. Although this prevalence is lower than that reported in several international cohorts, continued ophthalmologic screening remains essential because ROP continues to be one of the leading preventable causes of childhood visual impairment worldwide [24].

Necrotizing enterocolitis was diagnosed in 15.5% of patients and represented one of the most severe gastrointestinal complications observed. Although the annual frequency of necrotizing enterocolitis varied during the study period, no statistically significant temporal differences were observed. Given the limited number of cases, these findings should be interpreted descriptively and should not be considered evidence of a temporal trend. Nevertheless, improvements in nutritional management, increased use of human milk, and enhanced infection-prevention measures may have contributed to the observed distribution. Similar reductions have been reported following implementation of quality-improvement initiatives in neonatal intensive care units [14,22].

The relatively high incidence of NEC observed in our cohort should be interpreted cautiously. Only three infants developed complicated NEC requiring surgical intervention, whereas the remaining cases were classified as suspected NEC (Bell stage IA). Inclusion of these early-stage or suspected cases may have contributed to an apparent increase in NEC incidence compared with studies reporting only definite or advanced NEC. In addition, referral bias and the high proportion of extremely premature infants admitted to our tertiary center may have influenced the observed frequency.

Sepsis was the only morbidity showing statistically significant variation across years of admission, with the highest annual frequency observed in 2022 (7 of 11 infants, 63.6%). However, this finding should be interpreted cautiously because of the small annual sample sizes, the multiple comparisons performed, and the retrospective design of the study. Therefore, this observation should be considered exploratory and hypothesis-generating rather than conclusive. Future studies with larger multicenter cohorts are needed to determine whether this variation reflects true epidemiological changes or random fluctuations. Previous studies from Latin America, Asia, and multinational neonatal cohorts have consistently identified sepsis as a major contributor to morbidity, prolonged hospitalization, healthcare costs, and mortality among VLBW infants [9,10,11,16,18,19,20]. Although the factors underlying the increase observed in our institution cannot be determined from this retrospective analysis, changes in patient volume, staffing levels, infection-control practices, antimicrobial stewardship programs, and local epidemiological conditions may have played a role. These findings highlight the importance of continuous surveillance and infection-prevention strategies within NICUs.

Deaths occurred across different gestational age and birth weight categories. Although lower gestational age and lower birth weight are well-established risk factors for mortality in VLBW infants, the limited number of deaths in the present cohort precluded reliable assessment of mortality patterns according to these characteristics. Therefore, the observed distribution should be interpreted descriptively and not as evidence of specific mortality risk profiles [4,5,18,19,20]. Although the observed mortality rate was within the range reported by several neonatal cohorts, this estimate should be interpreted cautiously because local viability practices, referral patterns, and the exclusion of infants not admitted to intensive care may have influenced the observed rate.

Comparison with international neonatal networks provides useful context for interpreting our findings. However, meaningful comparisons are limited by substantial differences in case mix, gestational age distribution, viability thresholds, referral practices, healthcare resources, and study methodologies across institutions and countries. Therefore, the mortality rate observed in our cohort should be considered primarily as a descriptive estimate rather than as evidence of superior or equivalent outcomes compared with other neonatal populations.

Regarding specific morbidities, the frequency of bronchopulmonary dysplasia (26.9%) and necrotizing enterocolitis (15.5%) was within the range reported by international neonatal networks, although direct comparisons should be interpreted cautiously because of differences in case mix, gestational age distribution, survival rates, and diagnostic criteria [5,18,19,20]. The relatively low prevalence of severe intraventricular hemorrhage and retinopathy of prematurity observed in our cohort may reflect improvements in neonatal care practices, respiratory management, and screening programs. However, these findings should be confirmed in larger multicenter studies.

Notably, international collaborative networks such as NEOCOSUR, the Neonatal Research Network of Japan, and other national neonatal databases have demonstrated that continuous benchmarking, standardized quality-improvement initiatives, infection-control programs, and evidence-based clinical protocols are associated with progressive reductions in mortality and severe morbidity among VLBW infants [4,5,14,18,19,20]. Published data from Mexico, especially from northeastern regions, remain scarce. Survival rates reported in Mexican studies vary widely, ranging from 38% to 92%, depending on study period, level of neonatal care, and inclusion of extremely preterm or extremely low-birth-weight infants. Consequently, direct comparisons across studies should be interpreted cautiously. Therefore, the lack of regional epidemiological evidence underscores the significance of the present study, which contributes valuable contemporary data on the morbidity and mortality of VLBW infants in this setting. The establishment of similar collaborative surveillance strategies in Mexico could facilitate outcome monitoring, interinstitutional comparisons, and implementation of targeted interventions aimed at further improving neonatal outcomes.

Comparison of survivors and non-survivors did not reveal statistically significant differences in gestational age, birth weight, sex, antenatal corticosteroid exposure, surfactant administration, type of pregnancy, or mode of delivery. Although survivors exhibited slightly higher gestational age and birth weight, these differences were modest and did not reach statistical significance. The lack of statistically significant associations between these classical risk factors and mortality likely reflects the limited sample size and the small number of deaths observed in the cohort rather than the absence of a true biological effect. Numerous studies have consistently demonstrated that lower gestational age and lower birth weight are among the strongest predictors of mortality in VLBW infants, particularly among those born at the limits of viability and in resource-constrained settings [4,5,11,18,19,20].

The median Apgar score at 5 min did not differ significantly between survivors and non-survivors (8 [IQR 8–9] vs. 8 [IQR 4.5–9]; *p* = 0.493). Although no statistically significant difference was observed, larger studies are needed to clarify the potential prognostic value of the Apgar score in VLBW infants.

Previous investigations have identified low Apgar scores as markers of perinatal compromise and predictors of adverse neonatal outcomes, including mortality, severe respiratory disease, and neurodevelopmental impairment among preterm infants [6,17,19,20].

Exposure to antenatal corticosteroids and surfactant therapy was not significantly associated with survival. Antenatal corticosteroids are recognized as one of the most effective interventions for reducing respiratory distress syndrome, intraventricular hemorrhage, and neonatal mortality among preterm infants [4,5,18,19,20]. Similarly, surfactant replacement therapy has contributed substantially to improvements in respiratory outcomes and survival among VLBW neonates worldwide [4,5,6,18,19,20]. The absence of statistically significant differences between survivors and non-survivors in our cohort may be explained by the relatively frequent use of these interventions across the study population and by the limited number of mortality events available for comparison.

Likewise, neither multiple gestation nor cesarean delivery demonstrated a statistically significant relationship with mortality. Although multiple pregnancies are associated with increased risks of prematurity and low birth weight, their independent contribution to mortality remains controversial after adjustment for gestational age and neonatal condition [7,8]. The high cesarean delivery rate observed in both survivors and non-survivors likely reflects institutional management practices for high-risk pregnancies and may have contributed to the outcomes observed in this cohort, as reported in other contemporary neonatal studies [5,12,18].

Length of hospital stay was significantly shorter among non-survivors, reflecting the occurrence of death during the early neonatal period rather than representing an independent risk factor. Similar observations have been reported by neonatal networks in Japan, Taiwan, Singapore, and South America, where mortality among VLBW infants is concentrated within the first days or weeks of life, particularly among those with severe respiratory compromise, sepsis, or multiple organ dysfunction [4,6,14,18,19,20].

### 4.1. Clinical Implications

These findings support continued efforts to increase antenatal corticosteroid exposure, strengthen infection-prevention programs, optimize respiratory support strategies, and establish regional neonatal surveillance networks in Mexico. Particular attention should continue to be directed toward extremely preterm and VLBW infants, given their well-recognized vulnerability reported in the literature, while acknowledging that the present study was not powered to establish mortality risk profiles.

### 4.2. Strengths and Limitations

Strengths of this study include the five-year study period, the use of standardized NICU clinical protocols, and the detailed characterization of morbidity patterns in a tertiary referral center from a region of Mexico where published neonatal outcome data remain scarce.

Several limitations should be considered when interpreting our findings. First, the retrospective single-center design may limit the generalizability of the results to other institutions and populations. Second, the relatively small sample size and limited number of deaths reduced the statistical power available for subgroup comparisons, temporal trend analyses, and identification of factors associated with mortality. Consequently, observed differences between groups should be interpreted cautiously and regarded as exploratory rather than definitive. Third, important severity-of-illness indicators, including the Clinical Risk Index for Babies (CRIB), Score for Neonatal Acute Physiology II (SNAP-II), and Transport Risk Index of Physiologic Stability (TRIPS), were not routinely recorded during the study period and therefore could not be incorporated into the analyses. Fourth, local viability practices may have influenced mortality estimates. In our institution, infants born at <25 weeks of gestation and/or with a birth weight < 600 g generally receive comfort care because survival in this subgroup has historically been extremely poor. As these infants are not routinely admitted to the NICU or included in the institutional database, the mortality rate reported in this study may underestimate the overall mortality among all VLBW and extremely preterm infants born at our center. Fifth, detailed anthropometric measurements required to classify intrauterine growth restriction as symmetric or asymmetric were not consistently documented, precluding further characterization of fetal growth patterns. Finally, bronchopulmonary dysplasia was defined according to the NIH 2001 [21] criteria because these definitions were consistently applied in clinical practice during the study period. More recent classifications, such as the Jensen 2019 criteria [26], could not be retrospectively applied because detailed data regarding respiratory support modalities were not consistently available and some contemporary respiratory devices, including high-flow nasal cannula therapy, were not routinely used in our institution. Consequently, comparisons with studies using newer BPD definitions should be interpreted cautiously. Additionally, some clinical outcomes, including sepsis, pneumonia, and necrotizing enterocolitis, were defined according to routine clinical practice and therefore included both microbiologically confirmed and clinically suspected cases. In particular, Bell stage IA cases were included in the NEC analysis, and sepsis and pneumonia were not restricted to culture-confirmed diagnoses. Consequently, the reported frequencies of these morbidities may appear higher than those reported in studies using more stringent diagnostic definitions, which should be considered when interpreting our findings and making direct comparisons with other neonatal cohorts. Furthermore, the study was limited to short-term in-hospital outcomes, and long-term growth, neurodevelopmental, and post-discharge outcomes were not assessed.

Despite these limitations, the present study provides valuable regional information regarding morbidity patterns, mortality, and clinical outcomes among VLBW preterm neonates in northeastern Mexico. Future multicenter collaborative studies integrating standardized severity scores, comprehensive perinatal data, and longitudinal follow-up will be essential to identify modifiable risk factors and guide quality-improvement initiatives aimed at reducing morbidity and mortality among VLBW infants in Mexico and other middle-income settings.

## 5. Conclusions

Very-low-birth-weight (VLBW) preterm neonates remain a highly vulnerable population with a substantial burden of morbidity despite advances in neonatal intensive care. In this cohort, respiratory distress syndrome, apnea of prematurity, hyperbilirubinemia, and sepsis were the most frequently encountered complications, underscoring the persistent impact of respiratory and infectious diseases on short-term neonatal outcomes.

Deaths occurred mainly in the context of severe respiratory and systemic complications, including neonatal asphyxia, pulmonary hypertension, sepsis, shock, and multiple organ failure. Although no statistically significant predictors of mortality were identified, deaths occurred across different gestational age and birth weight categories. Given the limited number of mortality events, no firm conclusions can be drawn regarding mortality risk profiles, and larger multicenter studies are needed to better characterize factors associated with mortality in this population. The observed mortality rate fell within the range reported by other neonatal cohorts; however, comparisons should be interpreted cautiously because of differences in study populations, local viability practices, referral patterns, exclusion criteria, and the limited sample size and number of mortality events in the present study.

These findings emphasize the importance of optimizing prenatal care, increasing antenatal corticosteroid coverage, strengthening infection-prevention strategies, and maintaining evidence-based respiratory management practices. Furthermore, the establishment of regional neonatal surveillance systems and quality-improvement initiatives may facilitate ongoing benchmarking and contribute to further reductions in morbidity and mortality among VLBW infants.

Future multicenter studies with larger sample sizes, standardized severity assessment, and longitudinal follow-up are needed to better characterize predictors of adverse outcomes and to support the development of targeted interventions aimed at improving neonatal survival and reducing long-term morbidity among VLBW infants in Mexico and other middle-income settings.

## Figures and Tables

**Table 1:** Demographic Characteristics of the Neonates.

Variable	Mean/Median/*n*	SD/IQR/%
Gestational age (weeks)		
Overall	29.8	2.7
<26	3	5.2
26–28	19	32.8
29–31	16	27.6
>31	20	34.5
Birth weight (g)		
Overall	1109	238
500–750	4	6.9
750–999	12	20.7
1000–1249	23	39.7
1250–1500	19	32.8
Sex		
Female	32	55.2
Male	26	44.8
Apgar score, median (IQR)		
1 min	7	5–8
5 min	8	7–9
Endotracheal surfactant administration	31	53.4
Days of invasive mechanical ventilation, median (IQR)	1	0–6
Days of non-invasive ventilation, median (IQR)	2	0–4
Cumulative days of oxygen therapy, median (IQR)	9.5	1.7–30
Length of hospital stay (days), median (IQR)	51.5	36–71.75

Data are presented as mean ± standard deviation (SD), median (interquartile range [IQR]), or number (%), according to data distribution. Normality was assessed using the Shapiro–Wilk test.

**Table 2:** Maternal sociodemographic characteristics.

Variable	Mean/*n*	SD/%
Maternal age (years)		
Overall	30.1	8.8
<20	6	10.3
20–35	36	62.1
>35	16	27.6
Educational level		
Primary school	1	1.7
Secondary school	15	25.9
High school	14	24.1
University degree	28	48.3
Marital status		
Single	12	20.7
Married	22	37.9
Cohabiting (common-law union)	24	41.4
Antenatal corticosteroid administration		
Yes	33	56.9
No	25	43.1
Mode of delivery		
Vaginal delivery	2	3.4
Cesarean section	56	96.6
Type of pregnancy		
Singleton	37	63.8
Multiple	21	36.2
Mode of conception		
Spontaneous	46	79.3
Assisted reproduction	12	20.7

Data are presented as numbers, means, percentages, and standard deviations. SD: standard deviation.

**Table 3:** Morbidity and mortality recorded in the study population according to gestational age.

Morbidity	<26 Weeks n (%) (n = 3)	26–28 Weeks N (%) (N = 19)	29–31 Weeks N (%) (N = 16)	>31 Weeks n (%) (n = 20)	Total n (%)
Respiratory Distress Syndrome (RDS)	3 (100.0)	18 (94.7)	12 (75.0)	14 (70.0)	47 (81.0)
Apnea of Prematurity	3 (100.0)	17 (89.5)	4 (25.0)	18 (90.0)	42 (72.4)
Hyperbilirubinemia	3 (100.0)	15 (78.9)	8 (50.0)	14 (70.0)	40 (68.9)
Pneumonia	2 (66.7)	10 (52.6)	4 (25.0)	4 (20.0)	20 (34.5)
Sepsis	3 (100.0)	9 (47.4)	7 (43.8)	0 (0.0)	19 (32.7)
Patent Ductus Arteriosus (PDA)	0 (0.0)	6 (31.6)	4 (25.0)	5 (25.0)	15 (25.8)
Bronchopulmonary Dysplasia (BPD)	2 (66.7)	8 (42.1)	3 (18.8)	1 (5.0)	14 (26.9)
Necrotizing Enterocolitis (NEC)	0 (0.0)	3 (15.8)	5 (31.3)	1 (5.0)	9 (15.5)
Intraventricular Hemorrhage (IVH)	1 (33.3)	4 (21.1)	2 (12.5)	0 (0.0)	7 (12.0)
Retinopathy of Prematurity (ROP)	1 (33.3)	2 (10.5)	1 (6.3)	1 (5.0)	5 (8.6)
Congenital Heart Disease	0 (0.0)	0 (0.0)	0 (0.0)	2 (10.0)	2 (3.4)
Death	0 (0.0)	3 (15.8)	1 (6.3)	2 (10.0)	6 (10.3)

Data are presented as numbers and percentages. Percentages for gestational age categories were calculated using the number of neonates within each gestational age group as the denominator. Percentages for bronchopulmonary dysplasia were calculated using only infants who survived to 36 weeks’ postmenstrual age as the denominator.

**Table 4:** Morbidity and mortality according to birth weight category.

Morbidity	500–749 g n (%) (n = 4)	750–999 g n (%) (n = 12)	1000–1249 g n (%) (n = 23)	1250–1500 g n (%) (n = 19)	Total n (%)
Respiratory Distress Syndrome (RDS)	4 (100.0)	11 (91.7)	21 (91.3)	11 (57.9)	47 (81.0)
Apnea of Prematurity	4 (100.0)	11 (91.7)	17 (73.9)	10 (52.6)	42 (72.4)
Hyperbilirubinemia	3 (75.0)	9 (75.0)	17 (73.9)	11 (57.9)	40 (68.9)
Pneumonia	2 (50.0)	6 (50.0)	9 (39.1)	3 (15.8)	20 (34.5)
Sepsis	2 (50.0)	8 (66.7)	7 (30.4)	2 (10.5)	19 (32.7)
Patent Ductus Arteriosus (PDA)	0 (0.0)	6 (50.0)	4 (17.4)	5 (26.3)	15 (25.8)
Bronchopulmonary Dysplasia (BPD)	1 (25.0)	6 (50.0)	6 (26.1)	1 (5.3)	14 (26.9)
Necrotizing Enterocolitis (NEC)	0 (0.0)	2 (16.7)	3 (13.0)	4 (21.1)	9 (15.5)
Intraventricular Hemorrhage (IVH)	1 (25.0)	4 (33.3)	2 (8.7)	0 (0.0)	7 (12.0)
Retinopathy of Prematurity (ROP)	1 (25.0)	2 (16.7)	1 (4.3)	1 (5.3)	5 (8.6)
Congenital Heart Disease	0 (0.0)	0 (0.0)	0 (0.0)	2 (10.5)	2 (3.4)
Death	1 (25.0)	1 (8.3)	2 (8.7)	2 (10.5)	6 (10.3)

Abbreviations: RDS, respiratory distress syndrome; PDA, patent ductus arteriosus; BPD, bronchopulmonary dysplasia; NEC, necrotizing enterocolitis; IVH, intraventricular hemorrhage; ROP, retinopathy of prematurity. Data are presented as numbers (%). Percentages for birth weight categories were calculated using the number of neonates within each birth weight group as the denominator.

**Table 5:** Annual frequency of neonatal morbidities according to year of admission (2019–2023).

Morbidity	2019 n (%) (n = 16)	2020 n (%) (n = 8)	2021 n (%) (n = 11)	2022 n (%) (n = 11)	2023 n (%) (n = 12)	Total n (%)	*p*-Value *
Respiratory Distress Syndrome (RDS)	13 (81.3)	7 (87.5)	6 (54.5)	11 (100.0)	10 (83.3)	47 (81.0)	0.097
Apnea of Prematurity	10 (62.5)	6 (75.0)	10 (90.9)	8 (72.7)	8 (66.7)	42 (72.4)	0.575
Hyperbilirubinemia	13 (81.3)	3 (37.5)	9 (81.8)	6 (54.5)	9 (75.0)	40 (68.9)	0.139
Sepsis	2 (12.5)	5 (62.5)	3 (27.3)	7 (63.6)	2 (16.7)	19 (32.7)	0.014
Patent Ductus Arteriosus (PDA)	6 (37.5)	2 (25.0)	1 (9.1)	2 (18.2)	4 (33.3)	15 (25.8)	0.488
Intraventricular Hemorrhage (IVH)	1 (6.3)	2 (25.0)	0 (0.0)	2 (18.2)	2 (16.7)	7 (12.0)	0.419
Necrotizing Enterocolitis (NEC)	2 (12.5)	3 (37.5)	2 (18.2)	1 (9.1)	1 (8.3)	9 (15.5)	0.414
Bronchopulmonary Dysplasia (BPD) **	4 (25.0)	2 (25.0)	3 (27.3)	4 (36.4)	1 (8.3)	14 (26.9)	0.629
Retinopathy of Prematurity (ROP)	3 (18.8)	0 (0.0)	1 (9.1)	1 (9.1)	0 (0.0)	5 (8.6)	0.409

Data are presented as numbers and percentages. Percentages were calculated using the number of VLBW infants admitted during each study year as the denominator (2019, n = 16; 2020, n = 8; 2021, n = 11; 2022, n = 11; and 2023, n = 12). *p*-values were calculated using the Chi-square test or Fisher’s exact test, as appropriate. * indicates *p* < 0.05 and ** indicates *p* < 0.01. Percentages for bronchopulmonary dysplasia were calculated using only infants who survived to 36 weeks’ postmenstrual age as the denominator.

**Table 6:** Comparison of clinical characteristics between survivors and non-survivors.

Variable	Survivors (*n* = 52)	Non-Survivors (*n* = 6)	*p*-Value
Gestational age (weeks), mean ± SD	29.84 ± 2.73	29.58 ± 3.38	0.832
Birth weight (g), mean ± SD	1119.35 ± 227.99	1025.00 ± 325.07	0.362
Female sex, *n* (%)	29 (55.8)	3 (50.0)	0.788 *
Apgar score at 5 min, median (IQR)	8 (8–9)	8 (4.5–9)	0.493 †
Antenatal corticosteroids, *n* (%)	31 (59.6)	2 (33.3)	0.387 *
Surfactant administration, *n* (%)	27 (51.9)	4 (66.7)	0.675 *
Multiple pregnancy, *n* (%)	20 (38.5)	1 (16.7)	0.402 *
Cesarean delivery, *n* (%)	51 (98.1)	5 (83.3)	0.198 *
Length of hospital stay (days), median (IQR)	58.5 (42.7–74.7)	2.0 (1.0–13.5)	0.010 †

* Data are presented as mean ± standard deviation (SD), median (interquartile range [IQR]), or number (%), as appropriate. Continuous variables were compared using Student’s *t*-test or the Median test, whereas categorical variables were compared using the Chi-square test or Fisher’s exact test. Fisher’s exact test; † Median test.

## Data Availability

The data presented in this study are available from the corresponding author upon reasonable request. The data are not publicly available due to ethical and privacy restrictions involving patient confidentiality and institutional regulations regarding the use of clinical data.

## Abbreviations

The following abbreviations are used in this manuscript:

**Abbreviation**	**Definition**
BPD	Bronchopulmonary Dysplasia
CI	Confidence Interval
EOS	Early-Onset Sepsis
GA	Gestational Age
HRAEV	Hospital Regional de Alta Especialidad de Ciudad Victoria
IVH	Intraventricular Hemorrhage
NEC	Necrotizing Enterocolitis
NICU	Neonatal Intensive Care Unit
OR	Odds Ratio
PDA	Patent Ductus Arteriosus
RDS	Respiratory Distress Syndrome
ROP	Retinopathy of Prematurity
SD	Standard Deviation
VLBW	Very Low Birth Weight
WHO	World Health Organization
NS	Neonatal Sepsis
IQR	Interquartile Range
ICROP	International Classification of Retinopathy of Prematurity
LOS	Late-Onset Sepsis
